# A Call for Drug Therapies for the Treatment of Social Behavior Disorders in Dementia: Systematic Review of Evidence and State of the Art

**DOI:** 10.3390/ijms231911550

**Published:** 2022-09-30

**Authors:** Chiara Cerami, Giulia Perini, Andrea Panzavolta, Matteo Cotta Ramusino, Alfredo Costa

**Affiliations:** 1 IUSS Cognitive Neuroscience (ICoN) Center, University School for Advanced Studies IUSS, 27100 Pavia, Italy; 2 Cognitive Computational Neuroscience Research Unit, IRCCS Mondino Foundation, 27100 Pavia, Italy; 3 Unit of Behavioral Neurology and Center for Cognitive Disorders and Dementias (CDCD), IRCCS Mondino Foundation, 27100 Pavia, Italy; 4 Dementia Research Center (DRC), IRCCS Mondino Foundation, 27100 Pavia, Italy; 5 Department of Brain and Behavioral Sciences, University of Pavia, 27100 Pavia, Italy

**Keywords:** social cognition, pharmacological treatments, dementia, Alzheimer’s disease, neurocognitive disorder, frontotemporal dementia, oxytocin

## Abstract

Growing evidence supports the presence of social cognition deficits and social behavior alterations in major and minor neurocognitive disorders (NCDs). Even though the ability to identify socio-emotional changes has significantly improved in recent years, there is still no specific treatment available. Thus, we explored evidence of drug therapies targeting social cognition alterations in NCDs. Papers were selected according to PRISMA guidelines by searching on the PubMed and Scopus databases. Only papers reporting information on pharmacological interventions for the treatment of social cognition and/or social behavioral changes in major and/or minor NCDs were included. Among the 171 articles entered in the paper selection, only 9 papers were eligible for the scope of the review. Trials testing pharmacological treatments for socio-emotional alterations in NCDs are poor and of low-medium quality. A few attempts with neuroprotective, psychoactive, or immunomodulating drugs have been made. Oxytocin is the only drug specifically targeting the social brain that has been tested with promising results in frontotemporal dementia. Its beneficial effects in long-term use have yet to be evaluated. No recommendation can currently be provided. There is a long way to go to identify and test effective targets to treat social cognition changes in NCDs for the ultimate benefit of patients and caregivers.

## 1. Introduction

Social cognition is a multifaceted, complex domain encompassing every cognitive process aimed at recognizing and interpreting information acquired from the social environment, understanding one’s own or others’ behaviors, and modulating the way of thinking and acting according to the requirements of different social situations [1]. The latest version of the American Psychiatric Association’s Diagnostic and Statistical Manual for Mental Disorders (DSM-V) included social cognition among the neurocognitive domains that can be impaired in neurocognitive disorders (NCDs). Emotional blunting, lack of empathy or sympathy, and loss of social awareness, together with social disinhibition and socially inappropriate behaviors, usually characterize the clinical presentation of the behavioral variant of frontotemporal dementia (bvFTD) [2,3]. However, impairments in the theory of mind, empathy, or emotion recognition can be found not only in bvFTD, but also in other major NCDs, such as Alzheimer’s disease (AD) [4,5], and in minor NCDs, i.e., mild cognitive impairment (MCI) [6], suggesting that an impairment of social cognitive subdomains can be manifested even in a pre-dementia phase. In addition, elderly without dementia may also present deficits in social functioning due to mood changes, as proved in aged individuals with major depressive disorder (MDD), one of the most prevalent neuropsychiatric conditions among nursing homes resident without dementia [7,8,9]. In this group, social signals processing, reward/pain, and mentalizing disorders are predominant [9].

The management of socio-emotional changes has a huge effect on a patient’s family, caregivers, and the entire social network surrounding the patient. Both positive and negative social behavior changes significantly impact on caregiver and patient quality of life [10,11], affecting their social relationships [12] and causing burden and distress [13,14,15], with an inverse correlation between socio-emotional sensitivity scores and caregiver burden [16,17]. Despite this evidence, research studies on drug therapies for the treatment of social behavioral changes are poor, and there is no drug specifically targeting this condition actually available in clinical settings. Nonetheless, the control of social behavioral disorders may result in a double benefit, i.e., for patients and caregivers. Without treatments effectively targeting social cognition networks, physicians are unable to manage social behavioral symptoms, which can be deleterious and emotionally challenging to caregivers.

No effective disease-modifying treatments for neurocognitive disorders such as AD or frontotemporal dementia (FTD) are presently available, and symptomatic treatments for behavioral and/or cognitive disorders are the only way to manage the disease as the degeneration progresses. Current management of dementias thus includes non-pharmacologic treatments and symptomatic drugs (e.g., antidepressants or antipsychotics) able to control some cognitive and behavioral alterations. Non-pharmacologic treatments, such as psychosocial support and psychoeducational interventions for caregivers and physical, speech, and occupational therapy for patients, have proved of some utility in the management of behavioral symptoms [18,19,20]. None of these interventions, however, selectively target socio-emotional deficits.

The limited understanding of the biological bases of social cognition has been preventing for many years the development of selective pharmacological targets. The progress of imaging has helped in identifying the core structural and functional correlates of social functioning, with the limbic system (e.g., amygdala, insula, and cingulate cortex) as a primary hub within the so-called “social brain”, in close connection with the ventromedial prefrontal cortex, the superior temporal sulcus, and the temporo-parietal junction (see, for example, [21,22]). Many molecular effectors act within this complex architecture of interconnected brain regions.

Some molecules have gained more attention as they have been proven to be critical for social behavior. The efficacy of different drugs in controlling social behavior changes has been investigated in demented patients [23,24,25,26,27,28]. Oxytocin is certainly one of the most interesting and studied agents, as the amygdala is one of the core targets of oxytocin action in the brain [29]. This social peptide is produced in the supraoptic and paraventricular nuclei and stored in the neurohypophysis. The effects of intranasal administration of oxytocin are dependent on the presence and density of oxytocin receptors in target regions of social brain networks and have been tested using quantitative assessment of social cognition and emotion processing as clinical trial outcome measures [25,26,27,28]. Increased cooperative behavior, theory of mind, and empathy performance have been reported in volunteers [29,30,31,32,33,34]. Nonetheless, there is an open debate regarding prosocial effects of oxytocin [35,36,37]. Despite the abovementioned evidence of the positive effects of oxytocin treatment in humans [29,30,31,32,33,34], there is also proof that intranasal administration of oxytocin may have some negative social effects [35] and that this agent may influence the perception of non-social stimuli with strong personal relevance [36,37]. Moreover, the real-life long-term efficacy of oxytocin has been also questioned [38].

It remains an open question whether an effective pharmacological approach is a viable option to treat social cognition changes in neurocognitive patients. The aim of this review is thus to evaluate literature evidence on pharmacological interventions for the treatment of social cognition disorders (e.g., socially inappropriate behaviors and changes in the different facets of the social cognition domain) in NCD patients in order to disclose the lack of evidence and promote advances in pharmacological investigation.

## 2. Methods

The search process was performed by A.P., C.C., and G.P. in the PubMed and Scopus databases. The present study followed the PRISMA guidelines (see Tables S1 and S2 in the Supplementary Materials). Literature research was performed through search strings, with a combination of Medial Subject Headings (MeSH) and text word, including terms for pharmacological treatments, the different social cognition disorders, and the pathological condition of interest (i.e., (“drug therapy” OR “pharmacological intervention” OR “pharmacological treatment”) AND (“social cognition” OR “emotion recognition” OR “emotion processing” OR “empathy” OR “theory of mind” OR “social behavior” OR “social disinhibition” OR “social withdrawal” OR “social awareness” OR “social insight” OR “moral cognition” OR “social norms”) AND “neurocognitive disorders” OR “dementia*” OR “mild cognitive impairment” OR “Alzheimer*” OR “frontotemporal dementia”). Relevant references from previous personal knowledge and citation tracking articles were also included. Inclusion criteria for paper selection were: (a) full original papers (conference abstracts, case reports, reviews, and book chapters were excluded); (b) clinical trials in humans or animal models; (c) drugs targeting social behavior changes; and (d) language and time span (only papers published in English and up to May 2022).

**Figure 1:**
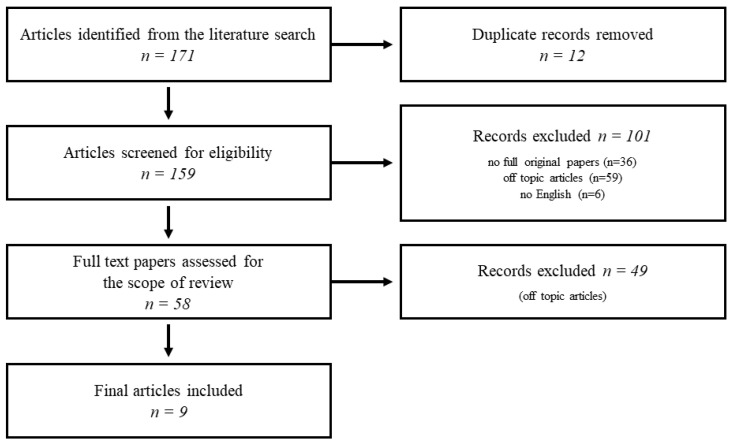
Flowchart of literature search.

The final records were uploaded to Rayyan, a free web and mobile app that helps expedite the initial screening of abstracts and titles using a process of semi-automation while incorporating a high level of usability [39]. Starting from the 171 papers initially included in the selection, we excluded 12 duplicates. Then, 101 articles were excluded after a first screening (36 articles were not full original papers, 59 were off-topic articles, 6 articles were not in the English language). Further, 49 articles were excluded after abstract or full text revision, because they were considered off-topic. Only nine papers were eventually considered eligible for the scope of the present review. Six papers reported results of clinical studies, while three were trials on animal models. See Figure 1 for details on the flow-chart of paper selection.

Finally, two authors (C.C. and A.P.) independently assessed study quality using the National Institutes of Health (NIH) Study Quality Assessment (SQA) tools [40] for the human studies and the Systematic Review Center for Laboratory animal Experimentation (SYRCLE) risk of bias tool [41] for preclinical studies.

## 3. Literature Search Results

### 3.1. Drugs Tested in Animal Models

The search strategy identified three papers reporting trials on animal models [42,43,44]. The first study by Zhang and Schluesener [42] evaluated a 10-day oral administration of histone deacetylase inhibitor MS-275, an anti-inflammatory and immunomodulatory pharmacological agent, in APP/PS1 mice, a transgenic mouse model of AD exhibiting a remarkable elevation of β-amyloid production associated with typical cognitive and behavioral changes. MS-275 is a class I histone deacetylase (HDAC) inhibitor that modulates epigenetic processes through hyperacetylation of histones and nonhistone proteins. HDACs play a pivotal role in cognitive processes and neurodegenerative diseases as well as in psychiatric disorders [45]. A correlation between reduced histone acetylation and cognitive or behavioral deficits in animal models of AD was previously demonstrated [46], supporting its use as treatment target. A nest construction assay was used to evaluate affiliative/social behavior performance in mice [47]. Study results indicated an improved nesting behavior in treated mice compared to non-treated ones. No data about safety issues were reported.

A more recent study from Castro et al. (2013) [43] tested a 7-day pre-treatment with oral atorvastatin (10 mg/kg/day) on social cognitive functioning in an MPTP mouse model of Parkinson’s disease (PD) suffering from early impairments in olfactory, cognitive, and socio-emotional functioning, and later presenting with motor dysfunctions. Statins are competitive inhibitors of 3-hydroxy-3-methylglutaryl coenzyme A (HMG-CoA) reductase with a specific role in cholesterol reduction and a pleiotropic neuroprotective, anti-inflammatory, and immunomodulating effect that have been proved to be beneficial on neurodegenerative disorders such as AD by reducing toxic Aβ amyloid plaques deposition and the aggregation of other proteins [48]. There is evidence from clinical and animal studies that statins might be beneficial as a therapy for cognitive deficits [49,50] and depressive-like behavior [51], although the exact mechanisms remain unclear. In a study by Castro et al. [43], treatment effect was evaluated with a short-term social recognition task as described by Dantzer and coworkers [52]. The pretreatment with atorvastatin prevented the deficit in social recognition ability induced in the PD model. Safety data were not reported.

Finally, Subramaniam and colleagues [44] evaluated the effects of a subcutaneous chronic administration of nicotine (0.4 mg/kg/h for one month) in a transgenic Thy1-aSyn mouse model of PD. Nicotine is a constituent of tobacco smoke that exerts psychoactive effects via binding to nicotinic acetylcholine receptors (nAChRs). Cholinergic deficits induced by α-synuclein pathology contribute to social impairments in PD. In this case, however, cholinesterase inhibitors have shown limited efficacy [53]. Treated mice presented cognitive and social behavior deficits with no motor impairments. Social cognition improvements were evaluated by means of the social approach task [54]. Both social behavior and cognition were significantly improved by nicotine administration. Mice receiving a nicotine dose of 0.4 mg/kg/h showed no differences in general health compared to mice administered with the vehicle.

See Table 1 for further details on the abovementioned studies.

Quality assessment of evidence for the included preclinical studies showed a middle-low overall quality. Results of the SYRCLE questionnaire have been reported in Table 1 as the rate of positive answers out of the total number of 10 SYRCLE questions. 

### 3.2. Drugs Tested in Clinical Trials

The search for eligible clinical trials (CTs) in NCD patients identified six studies [23,24,25,26,27,28]. A detailed description of each study is given on Table 1. Four studies were randomized CTs (RCTs) [24,25,26,28]; one study was an open CT [23]; and one described a novel design for a phase 2 clinical trial [27]. While studies from the 1980s and 1990s targeted vascular, mixed, or neurodegenerative dementias and enrolled participants from the general population or residents in long-term care facilities, since the 2000s researchers’ attention has been focused on the investigation of FTD patients in which social cognition dysfunctions are a core part of clinical presentation.

Blakemore [23] reported an open multicenter CT conducted by general practitioners in 303 elderly patients diagnosed with mild-to-moderate multi-infarct dementia and treated with cyclandelate 800 mg twice a day for 12 weeks. Cyclandelate is a vasodilator, with a direct-acting smooth muscle relaxant with a calcium-channel antagonism action. Effects on the central nervous system include ischemic protection, protection against hypoxia, decrease in hydroxyl radicals, inhibition of serotoninergic neurotransmission, improvement of oxygen exploitation and glucose utilization, increase of P300 amplitude in event-related cortical potentials, and stimulation of the memory and learning skills [55]. Primary outcome measures included global score and social cognition subscores (namely, social behavior and interest in others and in the environment) of the Parkside Behavioural Scale [56]. Post-treatment significant improvements in all mean scores were reported. Data on adverse events were not specifically described, but patient withdrawals for medical reasons and any dropouts were not reported.

Another paper [24] described a double-blind, placebo-controlled study in which memantine administration was compared to placebo assumption in 66 elderly patients with mild-to-moderate vascular, mixed, or neurodegenerative dementia. Patients randomized in the treatment group received memantine 10 mg (from day 1 to 3), 20 mg (from day 4 to 7), and 30 mg (from week 2 to 6). Memantine is an adamantine derivative and a non-competitive N-methyl-d-aspartate (NMDA) receptor antagonist. The NMDA receptor is known to play a pivotal role in synaptic transmission and synaptic plasticity. Primary outcomes included global score and socio-emotional subscales (i.e., assessing lack of drive, emotional disturbances, and social behavior) of the Sandoz Clinical Assessment Geriatric scale (SCAG) [57]. A post-treatment significant improvement of SCAG global and subscale scores was observed already after 14 days of memantine treatment in the treated group compared to the placebo group. The significant improvement was more pronounced at the end of the treatment period (after 6 weeks). Reported adverse drug effects were agitation/excitation, increased motor activity, sleeplessness, and restlessness, mainly at the beginning of the treatment, due to a too fast dose increase.

The most recent CTs identified by the literature research focused on oxytocin effects. Jesso et al. [25] presented the first randomized, double-blind, placebo-controlled, crossover study evaluating oxytocin effects in FTD. It was conducted in 20 patients fulfilling criteria for bvFTD [58] who received a single dose of 24 IU of intranasal oxytocin. The primary outcome measure was emotion recognition and processing, operationalized using the Facial Expression Recognition and Intensity task (modified from [59]), vocal affect recognition task [60], and the Mind in the Eyes task, and behavioral changes, identified with the Neuropsychiatric Inventory (NPI) [61] and the Frontal Behavioural Inventory (FBI) [62]. Oxytocin effects were evaluated at 8 h and one week following drug administration. A significant improvement of FTD-related neuropsychiatric behaviors as assessed with the NPI global score was found at 8 h in the treated group vs. the placebo group and compared with baseline ratings, but not after 1 week. The oxytocin group compared to the placebo group proved to have reduced identification of fearful and threatening stimuli, suggesting an increase of cooperative behavior after the neuropeptide administration. No significant effects on vocal affect recognition and poorer accuracy on the Mind in the Eyes task emerged after oxytocin administration. No significant adverse event was reported.

A subsequent study of Finger and coworkers [26] evaluated long-term administration of intranasal oxytocin. Finger et al. [26] conducted a randomized, parallel-group, double-blind, placebo-controlled study using a dose-escalation design to test the safety and tolerability of three clinically feasible doses of intranasal oxytocin (24, 48, or 72 IU) administered twice a day for one week in 23 patients with bvFTD or semantic dementia with concomitant behavioral disorders. Secondary outcomes explored efficacy across the treated vs. non-treated group. The NPI, FBI, Interpersonal Reactivity Index (IRI) [63], Frontotemporal Lobar Degeneration–modified Clinical Dementia Rating [64], and Frontotemporal Dementia Rating Scale [65] were used as outcome measures. A trend of improvement in NPI and FBI apathy scores, and the IRI empathic concern subscale score was observed with the administration of the maximum feasible dose (72 IU). The treatment was not significantly associated with adverse events or significant changes in positive NPI score, although an increase in hypersexual behaviors was reported.

A second study of Finger et al. [27] describes the design of a phase 2, adaptive, randomized, placebo-controlled, crossover trial. In stage 1, 60 FTD patients would receive three different dose schedules of 72 IU intranasal oxytocin (i.e., daily, alternate days, or every third day dosing) or placebo in order to identify the most promising dose scheduling; then, after a 6-week washout, patients would receive for 6 weeks the alternate drug (placebo or oxytocin). In stage 2, 40 additional patients would be enrolled in the most promising dose arm. The primary outcome would be NPI apathy/indifference domain score, while secondary outcomes would include IRI empathic concern subscale, NPI caregiver distress, and Revised Self-Monitoring Scale (i.e., rSMS [66]) scores and objective ratings of emotional facial expression and naturalistic videotaped behaviors in patients using the Social Observation Checklist [67]. Cerebrospinal fluid (CSF) oxytocin level would also be quantified as an outcome measure. In this paper [27], the authors concluded that the use of an adaptive crossover design and the inclusion of objective measures of socio-emotional performance and CSF oxytocin levels changes would help in improving RCT efficiency and conclusiveness.

Finally, the last study identified from the literature search [28] was a randomized, placebo-controlled, crossover study in 28 patients with bvFTD or a semantic variant of primary progressive aphasia (PPA) with social behavioral disorders who received a single dose of 72 IU intranasal oxytocin. Patients were asked to complete a functional MRI (fMRI) facial expression mimicry task and three behavioral tasks outside of the scanner (i.e., a short version of the View and Imitate Task, the Multifaceted Empathy Test (MET) [28,68,69], and the Postural Knowledge Test (PKT) [70]). The aim was to determine whether oxytocin administration alone or in combination with emotional mimicry training may increase brain activation. An increased fMRI activity was observed after oxytocin compared to placebo administration in fronto-limbic regions, key regions involved in emotion recognition and processing, and in the mentalizing network. The combination of oxytocin treatment and emotional mimicry training was associated with increased responses in these regions and in the right amygdala. Data on any adverse events were not reported.

The quality of evidence of human studies was evaluated in four papers [24,25,26,28] through the NIH SQA tool for Controlled Intervention Studies and in one paper [23] using the NIH SQA tool for Before-After (Pre-Post) Studies with No Control Group. Quality assessment was not performed for the paper of Finger and coworkers [27] describing a study design. Overall, the quality of evidence was middle-low. The results of the quality assessment in human studies have been reported in Table 1 as the percentage of positive answers out of the total number of SQA questions.

## 4. Discussion

NCDs are a very heterogeneous group of neurological conditions. Social cognition and behavior changes have been proved to be core symptoms in a relevant number of patients affected by different cognitive disorders [2,3,4,5,71,72]. Interest in the treatment of social cognition alterations has thus significantly increased in the last decades. This is particularly relevant in neurodegenerative conditions such as FTD in which socio-emotional deficits are a core part of the symptomatology [2]. Advances in technology have allowed for a better and earlier recognition of clinical manifestations and biological mechanisms of the different NCDs, so that molecular targets for novel pharmacological therapies have only recently been identified. Given that disease-modifying therapies are currently unavailable, efforts focused on the development of pharmacological and non-pharmacological treatments for the management of disabling symptoms in these patients should be promoted.

Our literature review shows that molecular approaches in animal models of dementia and clinical trials in patients with NCDs are limited in number and not yet conclusive. Attempts have been made by testing different drug targets in phase 1 studies on animal models of AD and PD, with inconsistent results on social cognition. Treatment with the immunomodulatory MS-275 drug improved nesting behavior in an APP/PS1 mouse model of AD as well as reduced neuroinflammation and Aβ deposition [42]. The use of atorvastatin in an MPTP mouse model of PD significantly enhanced social recognition memory, which is dependent on dopamine release, and prevented dopaminergic cell loss in the substantia nigra pars compacta. The authors, however, questioned whether both results may be, at least in part, due to an increase in NGF levels [43]. Chronic nicotine administration improved social behavior in a Thy1-aSyn mouse model of PD via direct stimulation of nAChRs. As suggested by the authors, in this case, a possible associated upregulation mechanism may have a role, while the neuroprotective effect on dopaminergic neurons remains unproven [44]. Each of the abovementioned drugs, however, showed single and not replicated evidence of efficacy in improving socio-emotional behavior and have not been further tested in humans.

Apart from a single open CT on cyclandelate and one RCT on memantine in vascular and neurodegenerative dementias showing some beneficial effects on social impairments, the most promising drug to treat social cognition deficits and social behavior changes in NCD patients is oxytocin. Both cyclandelate and memantine have general beneficial effects on the nervous system and do not specifically act on social cognition network functioning. Cyclandelate has been used in many European countries for over 20 years for the treatment of cerebral and peripheral vascular disease due to its neuroprotective effects [73]. Comparably, memantine received marketing authorization from the European Agency for the Evaluation of Medicinal Products (EMEA) for the treatment of moderately severe to severe AD in Europe in 2002 and shortly thereafter in the US by the Food and Drug Administration (FDA), because it has been shown to exert symptomatic and neuroprotective effects [74].

The identification of oxytocin as a neuropeptide specifically modulating social behavior across species has paved the way to the trials for the treatment of socio-emotional changes across neuropsychiatric conditions [75]. Healthy adults and patients with autism showed an improvement in emotional expression processing [76,77], empathy [34], and cooperative behavior [30] after the administration of oxytocin. The mechanisms accounting for these effects are somewhat unclear, although interactions across multiple neurotransmitter systems (e.g., dopaminergic and serotoninergic) are likely. Starting from this evidence, in 2011 the first RCT with intranasal oxytocin in FTD patients was performed. Oxytocin is produced in the supraoptic and paraventricular nuclei of the hypothalamus and released into circulation by the posterior pituitary. Both hypothalamic nuclei have been proved to be preserved in patients with TDP-43 (TAR DNA-binding protein 43) proteinopathy [78], suggesting possible differential responses to oxytocin treatments according to the underlying molecular pathologies. Oxytocin is also delivered directly to brain regions with afferent projections and paracrine signaling of oxytocin receptors, which are directly involved in emotion and reward processing [79,80]. These include the amygdala, medial prefrontal cortex, insula, and nucleus accumbens, which are particularly affected in FTD [81]. Evidence from the reviewed RCTs showed that a single dose of 24 IU intranasal oxytocin is associated with a transient improvement in social behavior [25]. A formal dose-finding study in FTD identified 72 IU twice a day as the most feasible dose scheduling [26]. and a single dose of 72 IU intranasal oxytocin is capable of increasing neural activity in affected brain regions during social cognition tasks [28]. Despite these promising preliminary beneficial effects with little or no reported side effects, evidence supporting the long-term use of oxytocin is crucially lacking to date.

Our study showed the relevant limitations of current evidence mainly related to a poor and heterogeneous body of literature using different designs, methodologies, and outcome measures to test possible drug targets. In addition, the poor/medium quality of the studies found by the literature search is a further limitation of the current literature and suggests the need for collecting additional high-quality evidence. Unique challenges for clinical trial design in the identification of effective, evidence-based symptomatic treatments for social cognition and behavior deficits in NCDs have previously been reported. First, like other behavioral disorders, socio-emotional symptoms exhibit a huge clinical heterogeneity, often with a non-linear trajectory over the course of the different NCD diseases and a strong dependence on the subjective reporting of caregivers [82]. Moreover, the use of different behavioral and cognitive measures to include cases and test treatment efficacy on social behavior or social cognition skills represents a crucial issue that prevents a real comparison among study findings. Harmonization on the use of social cognition measures across centers and CTs should be achieved together with the development of new personalized endpoints that are the most clinically meaningful to individuals and their families [83]. Goal attainment scaling (GAS) is an example of how a quantitative approach to measuring individual outcomes can be developed within a structured method for documenting patient-centered problems and care [84]. Additionally, the emerging correlation between behavioral deficits and available markers of neurodegeneration [85] highlights the need for more powerful analyses of combined clinical, genetic, imaging, and fluid biomarker data to improve the power to detect effective treatments, using a precision medicine approach [64]. Lastly, more widespread sharing of clinical trial data and biomarkers will be critical to developing new endpoints and to overcome sample size difficulties [86]. This is crucially relevant considering FTD research. As social dysfunction is a core clinical presentation of bvFTD and a common feature also in other FTD syndromes, critical issues also concern the rarity of these neurological conditions, the heterogeneity of clinical and neuropathological phenotypes, and the paucity of available biomarkers compared to more common diseases such as AD [86].

Finger and coworkers [27] focused in particular on additional challenges for RCTs of oxytocin, the main molecular target now available for social dysfunction treatment. These include neurochemicals issues to be addressed, i.e., potential differential responses according to sex, uncertainties around brain penetration of intranasal formulations, lack of dose-finding studies, and confirmation of target engagement [87]. Further, although some studies reported improvements in social cognition in several disorders following single-dose administration [32,33,76], longer-duration (e.g., 2–6 weeks) with once- or twice-daily dose schedule RCTs of oxytocin result in mixed findings, with null or small effects [88,89,90,91,92]. This might be due to the potential habituation of responses with chronic dosing that has been reported in animal studies [93,94,95,96]. With regard to the study design, Finger et al. [27] proposed an application of an adaptive crossover Bayesian design, in which the two-stage design allows at the same time the best dose schedule selection and efficacy assessment with a smaller sample size than traditional design. This turns to be particularly helpful when there are multiple goals in the trial. Moreover, the same authors highlighted the need to include in the trial design behavioral objective measures outcomes (i.e., videotaped naturalistic behaviors in addition to classic quantitative indirect measures relying on the caregiver judgments) and measurements of CSF oxytocin levels to confirm entry of the drug into the CNS. These represent valuable issues to be addressed to improve the efficiency and conclusiveness of future RCTs of oxytocin as well as other behavioral treatments in NCDs.

## 5. Conclusions

In conclusion, the management of socio-emotional changes in patients with NCDs is crucial, but the development and testing of effective drug therapies to improve social cognition is still a challenge. Systematic study of social cognitive functioning is currently lacking in clinical settings [97,98] and investigation of social cognition has only recently gained attention among the other neurocognitive domains that can be impaired in NCDs. For this reason, research studies investigating molecular mechanisms of social cognitive changes and exploring potential pharmacological targets for social behavior alterations in patients with NCDs are greatly lacking and those few that are available are not yet conclusive. At present, the only promising results in NCDs are those from the limited number of CTs testing oxytocin in FTD. According to this preliminary evidence, intranasal administration of oxytocin appears to be a possible avenue for the treatment of social cognitive disorders, at least in FTD. Nonetheless, administration issues and long-term efficacy need to be further investigated and oxytocin remains only promising approach that requires further and deeper investigation. Neuropathological heterogeneity in NCDs may also be relevant in view of the evaluation of oxytocin administration efficacy. Future studies need large-scale longitudinal investigations aimed at clarifying the role and molecular bases of social cognition changes in the different proteinopathies, according to the different pathological substrates not only in FTD but also in AD or α-synucleinopathies. This approach would certainly represent a boost to obtain more solid and reliable evidence that may overcome limitations of current studies in this field.

## Figures and Tables

**Table 1:** Pharmacological treatment studies in animal models and neurocognitive patients.

Authors	Year	Study Design	Sample Features	Treatment and Scheduling	Social Behavior and Cognition Measures	Study Findings	Quality Assessment
Zhang et al.	2013	Case-control study to test drug efficacy	6 treated APP/PS1-21 double transgenic mice 6 untreated APP/PS1-21 double transgenic mice 6 age- and sex-matched wild-type mice	10-day oral administration of a histone deacetylase inhibitor (MS-275)	A nest construction assay to evaluate affiliative/social behavior	Improved nesting behavior in treated mice compared to non-treated ones	Middle Quality (SYRCLE Score = 5/10)
Castro et al.	2013	Case-control study to test drug efficacy	Two independent cohorts of Wistar rats (63 adult and juvenile males; 34 treated with intranasal administration of MPTP to induce Parkinson’s symptomatology)	7-day pretreatment with oral atorvastatin (10 mg/kg/day)	Short-term social recognition task	Treatment with atorvastatin prevented the short-term social recognition memory impairments induced in the MPTP model	Middle Quality (SYRCLE Score = 5/10)
Subramaniam et al.	2018	Case-control study to test drug efficacy	35 Thy1-aSynuclein transgenic mice 41 wild-type mice	1 month of 0.4 mg/kg/h subcutaneous nicotine infusion	A social approach task	Improved social behavior in treated mice	Middle Quality (SYRCLE Score = 6/10)
Blakemore	1987	Open, multicenter clinical trial to test drug efficacy	303 patients with mild-to-moderate multi-infarct dementia (>65 years old)	12 weeks of oral administration of cyclandelate 1600 mg/day	Parkside Behavioural Rating Scale social cognition subscale	Improved social scores in treated patients	Low Quality (NIH Study QA Tool Score = 35%)
Ditzler	1991	Case-control study to test drug efficacy	66 mild to moderate dementia patients (43 female and 23 male; mean age 72 years old) randomized in treatment and control groups	Memantine 10 mg (from day 1 to 3), memantine 20 mg (from day 4 to 7), and memantine 30 mg (from week 2 to 6)	Sandoz Clinical Assessment Geriatric scale (SCAG) socio-emotional subscales	Improved socio-affective behavior changes in treated compared to placebo group (already after 14 days and more pronounced after 6 weeks)	Middle Quality (NIH Study QA Tool Score = 50%)
Jesso et al.	2011	Placebo-controlled study to test drug efficacy	20 bvFTD patients (64.4 ± 7.4 years old; 12.85 ± 3.3 years of education) randomized in treatment and placebo groups	Single dose of intranasal oxytocin (24 IU)	Facial Expression Recognition and Intensity task, vocal affect recognition task, Mind in the Eyes task	Significant reduced identification of anger and a trend of reduced fear recognition in treated vs. placebo group, and poorer Mind in the Eyes task accuracy in treated vs. placebo group after 20 min from drug administration. No significant effects of treatment after 2 weeks	Low Quality (NIH SQA Score = 40%)
Finger et al.	2015	Randomized, parallel-group, double-blind, placebo-controlled study to test drug safety and tolerability	23 FTD patients randomized in 3 dosage escalation treatment groups (61.1 mean years of age; 12.9 mean years of education) and a placebo group (66.0 mean years of age; 13.6 mean years of education)	1-week of intranasal oxytocin administration of 24, 48, or 72 IU twice a day	Interpersonal Reactivity Index (IRI)	Repeated doses of intranasal oxytocin are safe and well tolerated; after 72 IU, a trend towards an improvement of NPI and FBI apathy scores and IRI emphatic concern subscale	Middle Quality (NIH Study QA Tool Score = 60%)
Finger et al.	2018	Phase 2, adaptive, randomized, placebo-controlled, crossover trial to test dose-escalation design model	60 FTD patients (stage 1) 40 additional FTD patients (stage 2)	In stage 1, patients would receive three different dose schedules of 72 IU intranasal oxytocin (daily, alternate days, or every third day dosing) or placebo in order to identify the most promising dose scheduling; then, after 6-week washout, patients would receive for 6 weeks the alternate drug (placebo or oxytocin) In stage 2, 40 additional patients would be enrolled in the most promising dose arm	Interpersonal Reactivity Index (IRI) empathic concern subscale, Revised Self-Monitoring Scale (rSMS) and objective ratings of emotional facial expression and naturalistic videotaped behaviors in patients using the Social Observation Checklist (tested at baseline, at the end of stage 1, after wash-out, and at the end of stage 2)	Adaptive crossover design may facilitate oxytocin dose selection and efficacy assessment for symptomatic treatment of social disorders in FTD	NA
Olivero et al.	2020	Randomized, placebo-controlled, crossover study to test fMRI activation	28 FTD patients (including bvFTD and semantic variant PPA), mean age 64.29 (±7.88); 23 HC, mean age 61.39 (±7.04)	Single dose of intranasal oxytocin (72 IU)	View and Imitate Task, Multifaceted Empathy Test	Increased fMRI activity after oxytocin administration in treated compared to placebo group in fronto-limbic regions	Middle Quality (NIH Study QA Tool Score = 50%)

APP = amyloid precursor protein; PS1 = presenilin 1; MPTP = 1-methyl-4-phenyl-1,2,3,6-tetrahydropyridine; bvFTD = behavioral variant of frontotemporal dementia; FTD = frontotemporal dementia; PPA = primary progressive aphasia; fMRI = functional magnetic resonance imaging; SYRCLE = Systematic Review Center for Laboratory animal Experimentation; NIH = National Institutes of Health; QA = quality assessment; NA = not applicable.

## Data Availability

Not applicable.

## Supplementary Materials

The following supporting information can be downloaded at: https://www.mdpi.com/article/10.3390/ijms231911550/s1. Table S1. PRISMA guideline for abstract. Table S2. PRISMA guideline for reporting systematic reviews.

## References

[1] Frith C.D. (2008). Social cognition. Philos. Trans. R. Soc. Lond. B Biol. Sci..

[2] Cerami C., Cappa S.F. (2013). The behavioral variant of frontotemporal dementia: Linking neuropathology to social cognition. Neurol. Sci..

[3] Piguet O., Kumfor F. (2020). Frontotemporal dementias: Main syndromes and underlying brain changes. Curr. Opin. Neurol..

[4] Bora E., Walterfang M., Velakoulis D. (2015). Theory of mind in behavioural-variant frontotemporal dementia and Alzheimer’s disease: A meta-analysis. J. Neurol. Neurosurg. Psychiatry.

[5] Bora E., Velakoulis D., Walterfang M. (2016). Meta-Analysis of Facial Emotion Recognition in Behavioral variant frontotemporal dementia: Comparison with Alzheimer disease and healthy controls. J. Geriatr. Psychiatry Neurol..

[6] Bora E., Yener G.G. (2017). Meta-analysis of social cognition in Mild Cognitive Impairment. J. Geriatr. Psychiatry Neurol..

[7] Fornaro M., Solmi M., Stubbs B., Veronese N., Monaco F., Novello S., Fusco A., Anastasia A., De Berardis D., Carvalho A.F. (2020). Prevalence and correlates of major depressive disorder, bipolar disorder and schizophrenia among nursing home residents without dementia: Systematic review and meta-analysis. Br. J. Psychiatry J. Ment. Sci..

[8] Kim H.K., Nunes P.V., Oliveira K.C., Young L.T., Lafer B. (2016). Neuropathological relationship between major depression and dementia: A hypothetical model and review. Prog. Neuropsychopharmacol. Biol. Psychiatry.

[9] Porcelli S., Van Der Wee N., van der Werff S., Aghajani M., Glennon J.C., van Heukelum S., Mogavero F., Lobo A., Olivera F.J., Lobo E. (2019). Social brain, social dysfunction and social withdrawal. Neurosci. Biobehav. Rev..

[10] Diehl-Schmid J., Pohl C., Perneczky R., Förstl H., Kurz A. (2006). Behavioral disturbances in the course of frontotemporal dementia. Dement. Geriatr. Cogn. Disord..

[11] Riedijk S., Duivenvoorden H., Rosso S., Van Swieten J., Niermeijer M., Tibben A. (2008). Frontotemporal dementia: Change of familial caregiver burden and partner relation in a Dutch cohort of 63 patients. Dement. Geriatr. Cogn. Disord..

[12] Johnen A., Bertoux M. (2019). Psychological and cognitive markers of behavioral variant frontotemporal dementia-A clinical neuropsychologist’s view on diagnostic criteria and beyond. Front. Neurol..

[13] Boutoleau-Bretonnière C., Vercelletto M., Volteau C., Renou P., Lamy E. (2008). Zarit burden inventory and activities of daily living in the behavioral variant of frontotemporal dementia. Dement. Geriatr. Cogn. Disord..

[14] Mioshi E., Bristow M., Cook R., Hodges J.R. (2009). Factors underlying caregiver stress in frontotemporal dementia and Alzheimer’s disease. Dement. Geriatr. Cogn. Disord..

[15] Riedijk S.R., de Vugt M.E., Duivenvoorden H.J., Niermeijer M.F., van Swieten J.C., Verhey F.R.J., Tibben A. (2006). Caregiver burden, health-related quality of life and coping in dementia caregivers: A comparison of frontotemporal dementia and Alzheimer’s disease. Dement. Geriatr. Cogn. Disord..

[16] Mioshi E., Foxe D., Leslie F., Savage S., Hsieh S., Miller L., Hodges J.R., Piguet O. (2013). The impact of dementia severity on caregiver burden in frontotemporal dementia and Alzheimer disease. Alzheimer Dis. Assoc. Disord..

[17] Mourik J.C., Rosso S.M., Niermeijer M.F., Duivenvoorden H.J., Van Swieten J.C., Tibben A. (2004). Frontotemporal dementia: Behavioral symptoms and caregiver distress. Dement. Geriatr. Cogn. Disord..

[18] Gitlin L.N., Kales H.C., Lyketsos C.G. (2012). Nonpharmacologic management of behavioral symptoms in dementia. JAMA.

[19] Shnall A., Agate A., Grinberg A., Huijbregts M., Nguyen M.Q., Chow T.W. (2013). Development of supportive services for frontotemporal dementias through community engagement. Int. Rev. Psychiatry.

[20] Pressman P.S., Miller B.L. (2014). Diagnosis and management of behavioral variant frontotemporal dementia. Biol. Psychiatry.

[21] Bickart K.C., Dickerson B.C., Barrett L.F. (2014). The amygdala as a hub in brain networks that support social life. Neuropsychologia.

[22] Arioli M., Cattaneo Z., Ricciardi E., Canessa N. (2021). Overlapping and specific neural correlates for empathizing, affective mentalizing, and cognitive mentalizing: A coordinate-based meta-analytic study. Hum. Brain Mapp..

[23] Blakemore C.B. (1987). Cyclandelate in the treatment of multi-infarct dementia. Interim findings from a multicentre study in general practice. Drugs.

[24] Ditzler K. (1991). Efficacy and tolerability of memantine in patients with dementia syndrome. A double-blind, placebo-controlled trial. Arzneimittelforschung.

[25] Jesso S., Morlog D., Ross S., Pell M.D., Pasternak S.H., Mitchell D.G.V., Kertesz A., Finger E.C. (2011). The effects of oxytocin on social cognition and behaviour in frontotemporal dementia. Brain.

[26] Finger E.C., MacKinley J., Blair M., Oliver L.D., Jesso S., Tartaglia M.C., Borrie M., Wells J., Dziobek I., Pasternak S. (2015). Oxytocin for frontotemporal dementia: A randomized dose-finding study of safety and tolerability. Neurology.

[27] Finger E., Berry S., Cummings J., Coleman K., Hsiung R., Feldman H.H., Boxer A. (2018). Adaptive crossover designs for assessment of symptomatic treatments targeting behaviour in neurodegenerative disease: A phase 2 clinical trial of intranasal oxytocin for frontotemporal dementia (FOXY). Alzheimer’s. Res. Ther..

[28] Oliver L.D., Stewart C., Coleman K., Kryklywy J.H., Bartha R., Mitchell D.G.V., Finger E.C. (2020). Neural effects of oxytocin and mimicry in frontotemporal dementia: A randomized crossover study. Neurology.

[29] Meyer-Lindenberg A., Domes G., Kirsch P., Heinrichs M. (2011). Oxytocin and vasopressin in the human brain: Social neuropeptides for translational medicine. Nat. Rev. Neurosci..

[30] Kosfeld M., Heinrichs M., Zak P.J., Fischbacher U., Fehr E. (2005). Oxytocin increases trust in humans. Nature.

[31] Baumgartner T., Heinrichs M., Vonlanthen A., Fischbacher U., Fehr E. (2008). Oxytocin shapes the neural circuitry of trust and trust adaptation in humans. Neuron.

[32] Domes G., Heinrichs M., Gläscher J., Büchel C., Braus D.F., Herpertz S.C. (2007). Oxytocin attenuates amygdala responses to emotional faces regardless of valence. Biol. Psychiatry.

[33] Guastella A.J., Mitchell P.B., Dadds M.R. (2008). Oxytocin increases gaze to the eye region of human faces. Biol. Psychiatry.

[34] Hurlemann R., Patin A., Onur O.A., Cohen M.X., Baumgartner T., Metzler S., Dziobek I., Gallinat J., Wagner M., Maier W. (2010). Oxytocin enhances amygdala-dependent, socially reinforced learning and emotional empathy in humans. J. Neurosci..

[35] De Berardis D., Marini S., Iasevoli F., Tomasetti C., De Bartolomeis A., Mazza M., Valchera A., Fornaro M., Cavuto M., Srinivasan V. (2013). The role of intranasal oxytocin in the treatment of patients with schizophrenia: A systematic review. CNS Neurol. Disord. Drug Targets.

[36] Winterton A., Westlye L.T., Steen N.E., Andreassen O.A., Quintana D.S. (2021). Improving the precision of intranasal oxytocin research. Nat. Hum. Behav..

[37] Leng G., Leng R.I., Ludwig M. (2022). Oxytocin-a social peptide? Deconstructing the evidence. Philos. Trans. R. Soc. Lond. B Biol. Sci..

[38] Piguet O., Ahmed R.M., Kumfor F., Werry E.L., Reekie T.A., Kassiou M. (2022). The Role of Oxytocin in Social Circuits and Social Behavior in Dementia. Oxytocin, Methods and Protocols Methods in Molecular Biology.

[39] Ouzzani M., Hammady H., Fedorowicz Z., Elmagarmid A. (2016). Rayyan-a web and mobile app for systematic reviews. Syst. Rev..

[40] National Institutes of Health (2014). Quality Assessment Tool for Observational Cohort and Cross-Sectional Studies. https://www.nhlbi.nih.gov/health-pro/guidelines/in-develop/cardiovascular-risk-reduction/tools/cohort.

[41] Hooijmans C.R., Rovers M.M., de Vries R.B., Leenaars M., Ritskes-Hoitinga M., Langendam M.W. (2014). SYRCLE’s risk of bias tool for animal studies. BMC Med. Res. Methodol..

[42] Zhang Z.Y., Schluesener H.J. (2013). Oral administration of histone deacetylase inhibitor MS-275 ameliorates neuroinflammation and cerebral amyloidosis and improves behavior in a mouse model. J. Neuropathol. Exp. Neurol..

[43] Castro A.A., Wiemes B.P., Matheus F.C., Lapa F.R., Viola G.G., Santos A.R., Tasca C.I., Prediger R.D. (2013). Atorvastatin improves cognitive, emotional and motor impairments induced by intranasal 1-methyl-4-phenyl-1,2,3,6-tetrahydropyridine (MPTP) administration in rats, an experimental model of Parkinson’s disease. Brain Res..

[44] Subramaniam S.R., Magen I., Bove N., Zhu C., Lemesre V., Dutta G., Elias C.J., Lester H.A., Chesselet M.F. (2018). Chronic nicotine improves cognitive and social impairment in mice overexpressing wild type α-synuclein. Neurobiol. Dis..

[45] Abel T., Zukin R.S. (2008). Epigenetic targets of HDAC inhibition in neurodegenerative and psychiatric disorders. Curr. Opin. Pharmacol..

[46] Gräff J., Rei D., Guan J.S., Wang W.Y., Seo J., Hennig K.M., Nieland T.J.F., Fass D.M., Kao P.F., Kahn M. (2012). An epigenetic blockade of cognitive functions in the neurodegenerating brain. Nature.

[47] Wesson D.W., Wilson D.A. (2011). Age and gene overexpression interact to abolish nesting behavior in Tg2576 amyloid precursor protein (APP) mice. Behav. Brain Res..

[48] Bhat A., Dalvi H., Jain H., Rangaraj N., Singh S.B., Srivastava S. (2020). Perspective insights of repurposing the pleiotropic efficacy of statins in neurodegenerative disorders: An expository appraisal. Curr. Res. Pharmacol. Drug Discov..

[49] Jick H., Zornberg G.L., Jick S.S., Seshadri S., Drachman D.A. (2000). Statins and the risk of dementia. Lancet.

[50] Wolozin B., Wang S.W., Li N.C., Lee A., Lee T.A., Kazis L.E. (2007). Simvastatin is associated with a reduced incidence of dementia and Parkinson’s disease. BMC Med..

[51] Ludka F.K., Zomkowski A.D.E., Cunha M.P., Dal-Cim T., Zeni A.L.B., Rodrigues A.L.S., Tasca C.I. (2013). Acute atorvastatin treatment exerts antidepressant-like effect in mice via the L-arginine-nitric oxide-cyclic guanosine monophosphate pathway and increases BDNF levels. Eur. Neuropsychopharmacol..

[52] Dantzer R., Bluthe R.M., Koob G.F., Le Moal M. (1987). Modulation of social memory in male rats by neurohypophyseal peptides. Psychopharmacology.

[53] Wang H.F., Yu J.T., Tang S.W., Jiang T., Tan C.C., Meng X.F., Wang C., Tan M.S., Tan L. (2015). Efficacy and safety of cholinesterase inhibitors and memantine in cognitive impairment in Parkinson’s disease, Parkinson’s disease dementia, and dementia with Lewy bodies: Systematic review with meta-analysis and trial sequential analysis. J. Neurol. Neurosurg. Psychiatry.

[54] Magen I., Torres E.R., Dinh D., Chung A., Masliah E., Chesselet M.F. (2015). Social Cognition Impairments in Mice Overexpressing Alpha-Synuclein Under the Thy1 Promoter, a Model of Pre-manifest Parkinson’s Disease. J. Parkinsons Dis..

[55] Weyer G., Eul A., Milde K., Wierich W., Herrmann W.M. (2000). Cyclandelate in the treatment of patients with mild to moderate primary degenerative dementia of the Alzheimer type or vascular dementia: Experience from a placebo controlled multi-center study. Pharmacopsychiatry.

[56] Blakemore C.B., Stocker G. (1971). Improvement in certain aspects of behaviour of elderly patients treated by cyclandelate. Assessment in Cerebrovascular Insufficiency.

[57] Collegium Internationale Psychiatrie (CIPS) (1986). Sandoz Clinical Assessment Geriatric Scale.

[58] Rascovsky K., Hodges J.R., Knopman D., Mendez M.F., Kramer J.H., Neuhaus J., Van Swieten J.C., Seelaar H., Dopper E.G.P., Onyike C.U. (2011). Sensitivity of revised diagnostic criteria for the behavioural variant of frontotemporal dementia. Brain.

[59] Tottenham N., Tanaka J.W., Leon A.C., McCarry T., Nurse M., Hare T.A., Marcus D.J., Westerlund A., Casey B.J., Nelson C. (2009). The NimStim set of facial expressions: Judgments from untrained research participants. Psychiatry Res..

[60] Pell M.D., Paulmann S., Dara C., Alasseri A., Kotz S.A. (2009). Factors in the recognition of vocally expressed emotions: A comparison of four languages. J. Phon..

[61] Cummings J.L. (1997). The Neuropsychiatric Inventory: Assessing psychopathology in dementia patients. Neurology.

[62] Kertesz A., Davidson W., Fox H. (1997). Frontal behavioral inventory: Diagnostic criteria for frontal lobe dementia. Can. J. Neurol. Sci..

[63] Davis M.H. (1980). A multidimensional approach to individual differences in empathy. JSAS Catalog of Selected Documents in Psychology.

[64] Knopman D.S., Kramer J.H., Boeve B.F., Caselli R.J., Graff-Radford N.R., Mendez M.F., Miller B.L., Mercaldo N. (2008). Development of methodology for conducting clinical trials in frontotemporal lobar degeneration. Brain.

[65] Mioshi E., Hsieh S., Savage S., Hornberger M., Hodges J.R. (2010). Clinical staging and disease progression in frontotemporal dementia. Neurology.

[66] Lennox R.D., Wolfe R.N. (1984). Revision of the self-monitoring scale. J. Pers. Soc. Psychol..

[67] Mendez M.F., Fong S.S., Shapira J.S., Jimenez E.E., Kaiser N.C., Kremen S.A., Tsai P.H. (2014). Observation of social behavior in frontotemporal dementia. Am. J. Alzheimer’s Dis. Other Demen..

[68] Oliver L.D., Mitchell D.G., Dziobek I., MacKinley J., Coleman K., Rankin K.P., Finger E.C. (2015). Parsing cognitive and emotional empathy deficits for negative and positive stimuli in frontotemporal dementia. Neuropsychologia.

[69] Dziobek I., Rogers K., Fleck S., Bahnemann M., Heekeren H.R., Wolf O.T., Convit A. (2008). Dissociation of cognitive and emotional empathy in adults with Asperger syndrome using the Multifaceted Empathy Test (MET). J. Autism Dev. Disord..

[70] Mozaz M., Rothi L.J., Anderson J.M., Crucian G.P., Heilman K.M. (2002). Postural knowledge of transitive pantomimes and intransitive gestures. J. Int. Neuropsychol. Soc..

[71] Soleimani M.A.H., Negarandeh R., Bastani F., Grey R. (2014). Disrupted social connectedness in people with Parkinson’s disease. Br. J. Community Nurs..

[72] Setién-Suero E., Murillo-García N., Sevilla-Ramos M., Abreu-Fernández G., Pozueta A., Ayesa-Arriola R. (2022). Exploring the relationship between deficits in social cognition and neurodegenerative dementia: A systematic review. Front. Aging Neurosci..

[73] National Center for Biotechnology Information PubChem Compound Summary for CID 2893, Cyclandelate. https://pubchem.ncbi.nlm.nih.gov/compound/Cyclandelate#section=Literature.

[74] Rammes G., Danysz W., Parsons C.G. (2008). Pharmacodynamics of memantine: An update. Curr. Neuropharmacol..

[75] Insel T.R. (2010). The challenge of translation in social neuroscience: A review of oxytocin, vasopressin, and affiliative behavior. Neuron.

[76] Hollander E., Bartz J., Chaplin W., Phillips A., Sumner J., Soorya L., Anagnostou E., Wasserman S. (2007). Oxytocin increases retention of social cognition in autism. Biol. Psychiatry.

[77] Marsh A.A., Yu H.H., Pine D.S., Blair R.J. (2010). Oxytocin improves specific recognition of positive facial expressions. Psychopharmacology.

[78] Diodati D., Cyn-Ang L., Kertesz A., Finger E. (2012). Pathologic evaluation of the supraoptic and paraventricular nuclei in dementia. Can. J. Neurol. Sci..

[79] Loup F., Tribollet E., Dubois-Dauphin M., Dreifuss J.J. (1991). Localization of high-affinity binding sites for oxytocin and vasopressin in the human brain. An autoradiographic study. Brain Res..

[80] Boccia M.L., Petrusz P., Suzuki K., Marson L., Pedersen C.A. (2013). Immunohistochemical localization of oxytocin receptors in human brain. Neuroscience.

[81] Whitwell J.L., Josephs K.A. (2012). Recent advances in the imaging of frontotemporal dementia. Curr. Neurol. Neurosci. Rep..

[82] Miller J.B., Banks S.J., Leger G.C., Cummings J.L. (2014). Randomized controlled trials in frontotemporal dementia: Cognitive and behavioral outcomes. Transl. Neurodegener..

[83] Richardson E., Burnell J., Adams H.R., Bohannon R.W., Bush E.N., Campbell M., Chen W.H., Coons S.J., Papadopoulos E., Reeve B.R. (2019). Developing and implementing performance outcome assessments: Evidentiary, methodologic, and operational considerations. Ther. Innov. Regul. Sci..

[84] Kiresuk T.J., Sherman R.E. (1968). Goal attainment scaling: A general method for evaluating comprehensive community mental health programs. Community Ment. Health J..

[85] Cotta Ramusino M., Perini G., Vaghi G., Dal Fabbro B., Capelli M., Picascia M., Franciotta D., Farina L., Ballante E., Costa A. (2021). Correlation of frontal atrophy and CSF tau levels with neuropsychiatric symptoms in patients with cognitive impairment: A memory clinic experience. Front. Aging Neurosci..

[86] Boxer A.L., Gold M., Feldman H., Boeve B.F., Dickinson S.L., Fillit H., Ho C., Paul R., Pearlman R., Sutherland M. (2020). New directions in clinical trials for frontotemporal lobar degeneration: Methods and outcome measures. Alzheimer’s Dement..

[87] Insel T.R. (2016). Translating Oxytocin Neuroscience to the Clinic: A National Institute of Mental Health perspective. Biol. Psychiatry.

[88] Einfeld S.L., Smith E., McGregor I.S., Steinbeck K., Taffe J., Rice L.J., Horstead S.K., Rogers N., Hodge M.A., Guastella A.J. (2014). A double-blind randomized controlled trial of oxytocin nasal spray in Prader Willi syndrome. Am. J. Med. Genet. A.

[89] Yatawara C.J., Einfeld S.L., Hickie I.B., Davenport T.A., Guastella A.J. (2016). The effect of oxytocin nasal spray on social interaction deficits observed in young children with autism: A randomized clinical crossover trial. Mol. Psychiatry.

[90] Cacciotti-Saija C., Langdon R., Ward P.B., Hickie I.B., Scott E.M., Naismith S.L., Moore L., Alvares G.A., Redoblado Hodge M.A., Guastella A.J. (2015). A double-blind randomized controlled trial of oxytocin nasal spray and social cognition training for young people with early psychosis. Schizophr. Bull..

[91] Gibson C.M., Penn D.L., Smedley K.L., Leserman J., Elliott T., Pedersen C.A. (2014). A pilot six-week randomized controlled trial of oxytocin on social cognition and social skills in schizophrenia. Schizophr. Res..

[92] Anagnostou E., Soorya L., Chaplin W., Bartz J., Halpern D., Wasserman S., Wang A.T., Pepa L., Tanel N., Kushki A. (2012). Intranasal oxytocin versus placebo in the treatment of adults with autism spectrum disorders: A randomized controlled trial. Mol. Autism.

[93] Bales K.L., Perkeybile A.M., Conley O.G., Lee M.H., Guoynes C.D., Downing G.M., Yun C.R., Solomon M., Jacob S., Mendoza S.P. (2013). Chronic intranasal oxytocin causes long-term impairments in partner preference formation in male prairie voles. Biol. Psychiatry.

[94] Huang H., Michetti C., Busnelli M., Managò F., Sannino S., Scheggia D., Giancardo L., Sona D., Murino V., Chini B. (2014). Chronic and acute intranasal oxytocin produce divergent social effects in mice. Neuropsychopharmacology.

[95] Conti F., Sertic S., Reversi A., Chini B. (2009). Intracellular trafficking of the human oxytocin receptor: Evidence of receptor recycling via a Rab4/Rab5 “short cycle”. Am. J. Physiol. Endocrinol. Metab..

[96] Peters S., Slattery D.A., Uschold-Schmidt N., Reber S.O., Neumann I.D. (2014). Dose-dependent effects of chronic central infusion of oxytocin on anxiety, oxytocin receptor binding and stress-related parameters in mice. Psychoneuroendocrinology.

[97] Van den Stock J. (2022). Social cognition assessment for mild neurocognitive disorders. Alzheimer’s Dement..

[98] Dodich A., Boccardi M., Sacco L., Monsch A.U., Démonet J.F., Filardi M., Logroscino G., Salmon D.P., Weinbtraub S., Dubois B. (2022). Consortium for the Harmonization of Neuropsychological Assessment for Neurocognitive Disorders. Answer to “Social cognition assessment for mild neurocognitive disorders”. Alzheimer’s Dement..

